# Selective Growth of van der Waals Heterostructures Enabled by Electron-Beam Irradiation

**DOI:** 10.1021/acsami.3c02892

**Published:** 2023-07-07

**Authors:** Jakub Sitek, Karolina Czerniak-Łosiewicz, Arkadiusz P. Gertych, Małgorzata Giza, Paweł Dąbrowski, Maciej Rogala, Konrad Wilczyński, Anna Kaleta, Sławomir Kret, Ben R. Conran, Xiaochen Wang, Clifford McAleese, Michał Macha, Aleksandra Radenović, Mariusz Zdrojek, Iwona Pasternak, Włodek Strupiński

**Affiliations:** † Faculty of Physics, Warsaw University of Technology, Koszykowa 75, 00-662 Warsaw, Poland; ‡ CENTERA Laboratory, Institute for High Pressure Physics, Polish Academy of Sciences, Sokołowska 29, 01-142 Warsaw, Poland; § Faculty of Physics and Applied Informatics, University of Łódź, Pomorska 149/153, 90-236 Łódź, Poland; ∥ Institute of Physics, Polish Academy of Sciences, Al. Lotników 32/46, 02-668 Warsaw, Poland; ⊥ AIXTRON Ltd, Buckingway Business Park, Anderson Road, Swavesey, Cambridge CB24 4FQ, U.K.; # Laboratory of Nanoscale Biology, Swiss Federal Institute of Technology Lausanne (EPFL), Station 17, CH-015 Lausanne, Switzerland

**Keywords:** chemical vapor deposition, van der Waals heterostructures, electron-beam irradiation, selective growth, graphene

## Abstract

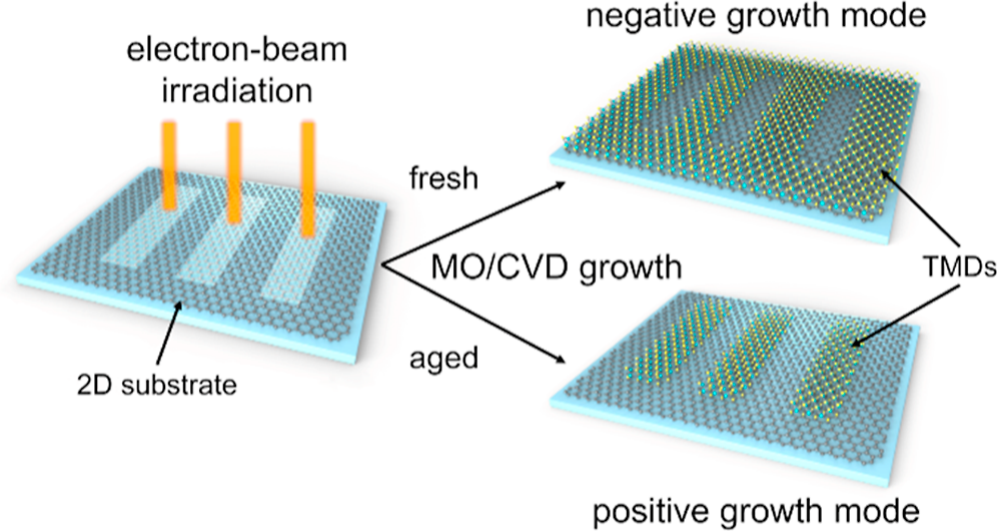

Van der Waals heterostructures (vdWHSs) enable the fabrication of complex electronic devices based on two-dimensional (2D) materials. Ideally, these vdWHSs should be fabricated in a scalable and repeatable way and only in the specific areas of the substrate to lower the number of technological operations inducing defects and impurities. Here, we present a method of selective fabrication of vdWHSs via chemical vapor deposition by electron-beam (EB) irradiation. We distinguish two growth modes: positive (2D materials nucleate on the irradiated regions) on graphene and tungsten disulfide (WS_2_) substrates, and negative (2D materials do not nucleate on the irradiated regions) on the graphene substrate. The growth mode is controlled by limiting the air exposure of the irradiated substrate and the time between irradiation and growth. We conducted Raman mapping, Kelvin-probe force microscopy, X-ray photoelectron spectroscopy, and density-functional theory modeling studies to investigate the selective growth mechanism. We conclude that the selective growth is explained by the competition of three effects: EB-induced defects, adsorption of carbon species, and electrostatic interaction. The method here is a critical step toward the industry-scale fabrication of 2D-materials-based devices.

## 1 Introduction

Two-dimensional (2D) materials stacked in the van der Waals heterostructures (vdWHSs) provide a unique playground for studying fundamental physics. Among the most substantial breakthroughs enabled by 2D materials stacks, one can indicate the discovery of unconventional superconductivity in magic-angle twisted bilayer graphene^1,2^ and other graphene stacks,^3,4^ high thermal anisotropy of van der Waals heterostructures,^5,6^ or existence of Wigner crystals^7^ and moiré trions^8^ in transition metal dichalcogenides (TMDs) stacks. vdWHSs enable not only research in fundamental physics but also in practical applications, especially in electronics. For example, stacking different 2D materials allows the fabrication of non-volatile flash memory,^9^ light-emitting diodes,^10^ or gas sensors.^11^

However, most 2D-based devices are fabricated using exfoliated and transferred 2D materials, which hinders their practical applications. Exfoliation induces defects and contamination in the layers,^12−14^ limiting the performance of the created electronic devices. Furthermore, exfoliation cannot be scaled to satisfy the needs of the electronics industry; hence, a repeatable and scalable method of fabricating vdWHSs is necessary. Among different synthesis methods, chemical vapor deposition (CVD) has already proved to yield transfer-free heterostructures of two or more different 2D materials aligned vertically or laterally.^15−17^ Notably, vertically-aligned stacks are preferable for electronic applications due to the more robust electrical contact between layers.^18,19^

Ideally, the area of the substrate where the 2D materials are synthesized should be easily controlled, as it limits the number of subsequent technological operations, including photopolymer spin-coating and chemical etching, and preserves the pristine surface. To date, selective growth of 2D materials has been achieved for single 2D materials^20^ and van der Waals heterostructures.^21^ Initially, selective growth was enabled by coating the substrate with photopolymer and subsequent deposition of seeds^22,23^ or modification of the substrate by partial etching.^24,25^ However, these methods require photolithographic pretreatment of the substrate, which provides little advantage compared to etching a complete 2D monolayer. More recent approaches include laser etching of 2D substrates, enabling the growth of vertical^26^ and lateral^27^ vdWHSs, or He focused-ion-beam-induced defects in graphene serving as controllable nucleation sites.^28^ Still, these methods heavily damage the substrate or need an elaborate experimental setup. An interesting approach is proposed by Ryu et al., showing a triboelectric-based method, which, however, significantly limits the shape of the selective growth area.^29^

In this work, we present a selective growth method based on electron-beam (EB) irradiation of 2D substrates. By irradiating a graphene substrate with an electron dose no lower than 5000 μC/cm^2^, we achieved highly selective growth of molybdenum disulfide (MoS_2_) and tungsten disulfide (WS_2_) in the irradiated areas, both by metal–organic and powder-based CVD. The selective growth of MoS_2_ was also achieved on the WS_2_ substrate, and we observed graphene self-healing after the subsequent TMDs synthesis.

Furthermore, we are able to block nucleation in the irradiated areas with 100 nm spatial resolution by limiting the substrate air exposure and time between irradiation and growth. These two growth modes are referred to as “positive” and “negative,” respectively, in a manner similar to the photoresist types. To investigate the selective growth model, we conducted Raman mapping, Kelvin-probe force microscopy (KPFM), X-ray photoelectron spectroscopy (XPS), and density-functional theory (DFT) modeling studies. Three competing mechanisms cause the observed effects: mild degradation of the 2D substrate, adsorption of carbon species during EB irradiation and air exposure, and electrostatic influence of the charged substrate. Considering that the presented method allows self-healing of graphene, provides exceptional spatial resolution, and enables control of the positive and negative growth, it is foreseen to revolutionize the industrial-scale growth of van der Waals heterostructures for electronics.

## 2 Results

### 2.1 Substrate Electron-Beam Irradiation

**Figure 1:**
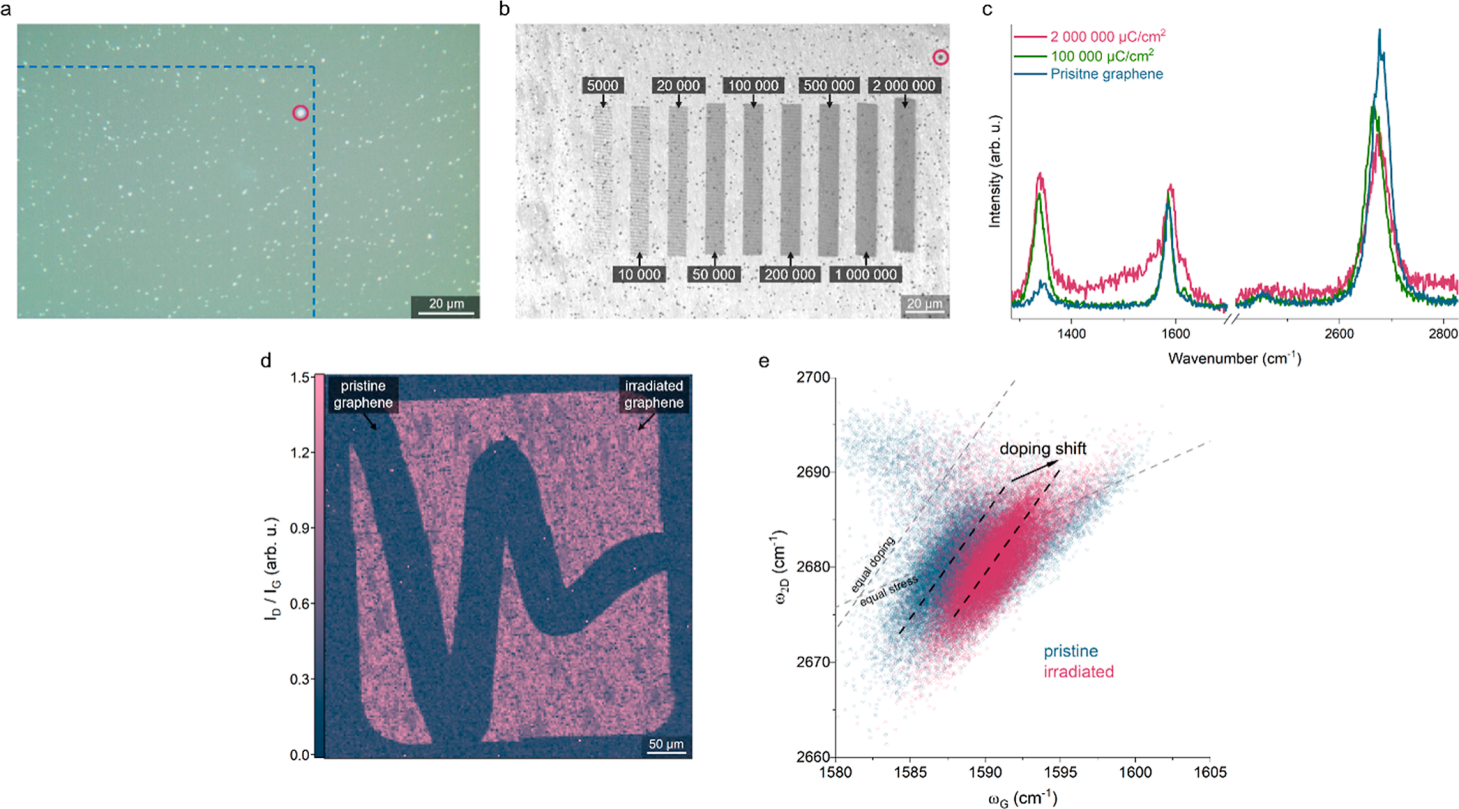
EB irradiation of the CVD graphene on sapphire. (a) Optical micrograph showing lack of contrast between irradiated and pristine graphene. The dashed blue lines indicate the boundary of the corresponding SEM image, shown in (b). (b) SEM image in in-lens contrast of the same area as in (a), displaying differences in work function between regions irradiated with different electron doses. The numbers above the stripes indicate the electron doses in μC/cm^2^. The stripe irradiated with the lowest applied dose, 1000 μC/cm^2^, cannot be distinguished. The same graphene defect is marked with a red circle in (a,b). (c) Raman spectra of pristine graphene and graphene irradiated with 100,000 and 2,000,000 μC/cm^2^. (d) The spatial mapping of the ratio of the D and G peaks integrated area displaying the distinct differences between graphene irradiated with 10,000 μC/cm^2^ and pristine regions. (e) Raman strain-doping decomposition of the area shown in (d). The irradiated areas are n-type doped but unstrained compared to pristine graphene.

For the selective growth, CVD graphene on sapphire was irradiated with electron doses varying between 1000 and 2,000,000 μC/cm^2^ (Figure S1). As presented in Figure 1a, the optical micrograph does not show any signs of degradation of the EB-irradiated graphene. However, the differences in work function on the irradiated areas are visible in in-lens imaging in scanning electron microscopy (SEM),^30^ but practically indistinguishable in secondary electrons imaging (Figures 1b and S2). Notably, by using EB lithography, it is possible to irradiate large areas with high spatial resolution up to 100 nm (Figure S3).

Raman spectra show that graphene is moderately degraded, expressed by a more intense D peak (Figure 1c, see also Figure S4), albeit the spectra confirm the presence of graphene structure, without graphene oxide^31^ or amorphous carbon.^32^ The difference in the ratio of D and G peaks intensity between pristine and irradiated regions is clear, as shown in a large Raman spatial mapping (Figure 1d, see also Figure S5). Furthermore, the Raman spatial mapping enabled us to deconvolute strain and doping in the irradiated areas.^33^ As presented in Figure 1e, the strain in graphene after irradiation has not changed, in contrast to doping, which indicates the *n*-type.

### 2.2 Positive Selective Growth

The selective growth of MoS_2_ and WS_2_ on the irradiated graphene substrates (both CVD and transferred) has been performed mainly in a tube furnace using molybdenum trioxide (MoO_3_), tungsten trioxide (WO_3_), and sulfur powders. Still, to prove the versatility of our method, some of the growth processes were conducted in a metal–organic CVD (MOCVD) system with molybdenum hexacarbonyl (Mo(CO)_6_) and diethyl sulfide (DES) as precursors. Our experiments indicated that it is necessary to age the irradiated substrates by exposing them to ambient air for at least 24 h to achieve positive growth. The aging process can be enhanced by mild annealing of the samples on a hot plate set to 70 °C.

**Figure 2:**
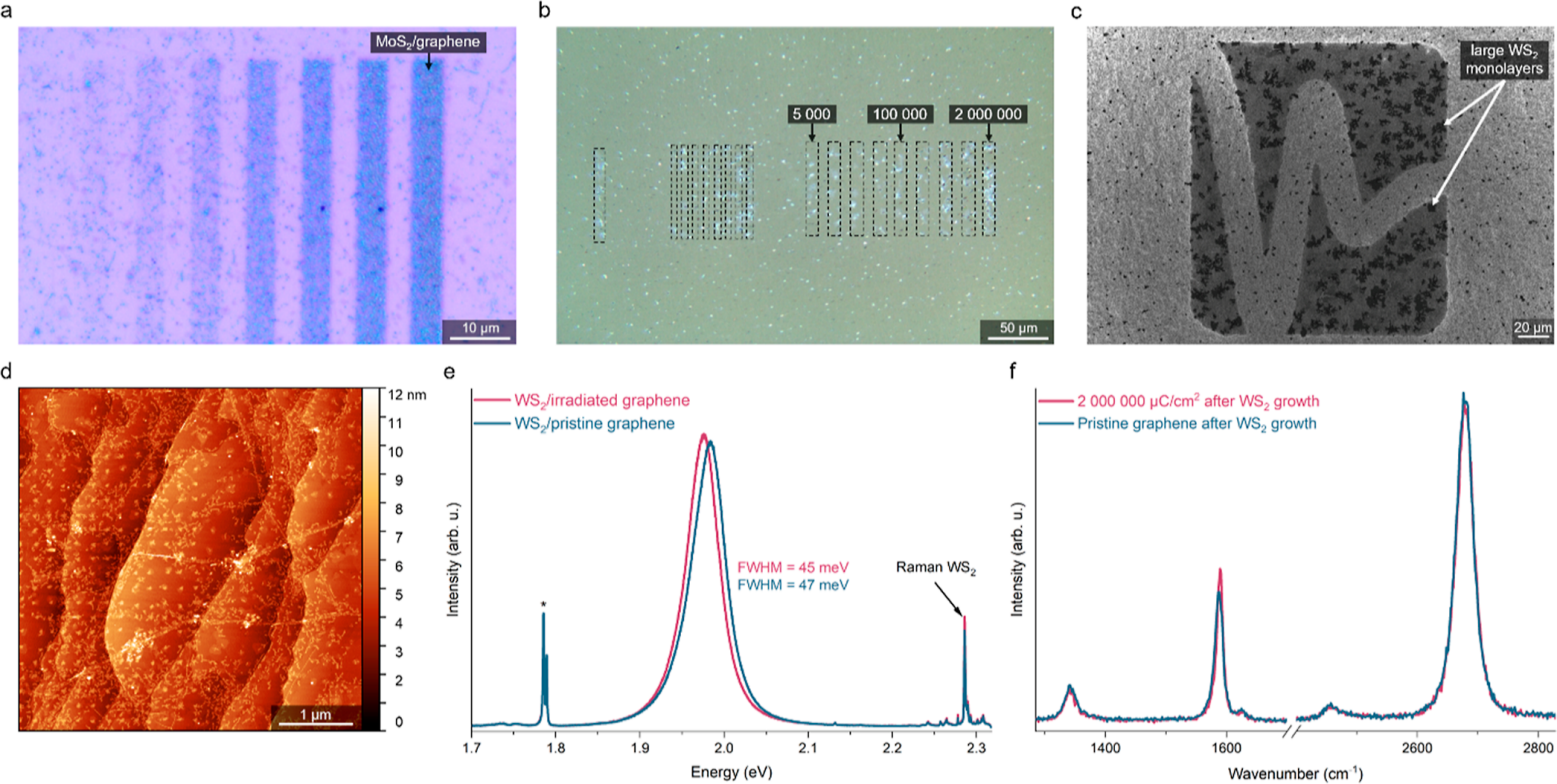
Selective positive growth of van der Waals heterostructures by EB irradiation on graphene transferred on SiO_2_/Si and grown by CVD on sapphire. (a) Optical micrograph of MoS_2_ synthesized on the irradiated transferred graphene. Higher nucleation occurs in the regions irradiated with higher electron doses. (b) Optical micrograph of WS_2_ synthesized on CVD graphene. The electron doses are given in μC/cm^2^. (c) SEM in-lens image of WS_2_ grown by CVD on irradiated CVD graphene. The change in work function is preserved after the WS_2_ growth. The large WS_2_ domains with irregular shapes nucleated preferentially on the irradiated areas. (d) AFM image of MoS_2_ grown by MOCVD on CVD graphene. MoS_2_ domains started nucleating in a 200 × 200 nm matrix determined by the translational step of the focused electron beam. (e) Photoluminescence of WS_2_ synthesized on CVD graphene. There is no significant difference in intensity and FWHM of WS_2_ synthesized on pristine and EB-irradiated graphene. A double peak originating from chromium impurities in sapphire is marked with an asterisk. (f) Self-healing of CVD graphene after WS_2_ growth. The Raman spectrum of graphene irradiated with the maximum dose (2,000,000 μC/cm^2^) after WS_2_ growth is identical to the spectrum of pristine graphene, in stark contrast with the spectrum obtained before the growth (Figure 1c).

The positive growth of MoS_2_ and WS_2_ is highly selective, as presented in Figure 2a–c. MoS_2_ (Figure 2a) nucleated predominantly on the irradiated areas of graphene transferred onto silicon dioxide (SiO_2_), and the nucleation is higher in the areas irradiated with higher electron dose. Notably, due to a higher number of defects (both impurities and wrinkles), higher nucleation is expected on transferred than CVD-grown samples. Furthermore, MoS_2_ nucleated more readily on irradiated WS_2_/graphene heterostructure (Figure S6). It proves that besides graphene, also WS_2_ and probably other 2D materials can be used as a substrate for selective growth enabled by electron beam irradiation.

Interestingly, the preferential growth of MoS_2_ on WS_2_/graphene presented in Figure S6 was achieved not by EB lithography but by an ordinary SEM imaging performed on a sample that was kept in an SEM chamber at 10^–6^ mbar for 16 h prior to characterization. One possible explanation is that during the 16 h period, carbon species from the chamber deposited onto the surface, and the imaging electron beam, much less intense than the typical EB irradiation used throughout the study, was able to cause the selective growth. Other samples characterized by SEM that were not kept in the chamber prior to characterization did not exhibit the selective growth effect.

Similar to MoS_2_, WS_2_ nucleated preferentially in the graphene areas irradiated with higher doses (Figure 2b). The minimum dose required to observe the positive growth effect is 5000 μC/cm^2^ (Figure 2b); however, the optimal value is between 10,000 and 1,000,000 μC/cm^2^. SEM image in in-lens contrast shows that the change in work function is preserved and confirms the preferred growth of large WS_2_ crystals on the irradiated areas (Figure 2c). MOCVD growth of MoS_2_ on CVD graphene on sapphire (Figures 2d and S7) indicated that nucleation starts directly on the irradiated spots, visible as a matrix of domains separated by 200 nm—the distance between irradiation points. Similar results obtained on CVD-grown and transferred graphene indicate that the supporting material below the 2D substrate (SiO_2_ or sapphire in our study) does not influence selective growth.

High-resolution transmission electron microscopy (HRTEM) images and energy-dispersive X-ray spectroscopy (EDX) confirmed that monolayer WS_2_ was synthesized on monolayer graphene (Figure S8). The interface is atomically sharp, with no contamination visible. The monolayered nature of the synthesized WS_2_ is also confirmed by PL measurements (Figure 2e). There is no significant difference in intensity and full width at half maximum (FWHM) of the WS_2_ photoluminescence (PL) peak on irradiated and pristine graphene. Thus, the irradiation does not influence the subsequent growth of other 2D materials. The uniformity of PL intensity of the synthesized WS_2_ is also excellent, as shown in Figure S9. Interestingly, the Raman spectrum of irradiated graphene does not show signs of degradation (as in Figure 1c) but rather self-healing, with a spectrum identical to the non-irradiated graphene after the growth process (Figures 2f and S10). In addition, atomic force microscopy (AFM) images prove that graphene is not degraded after the growth process (Figure S11). The proposed mechanism of self-healing is discussed later.

### 2.3 Negative Selective Growth

**Figure 3:**
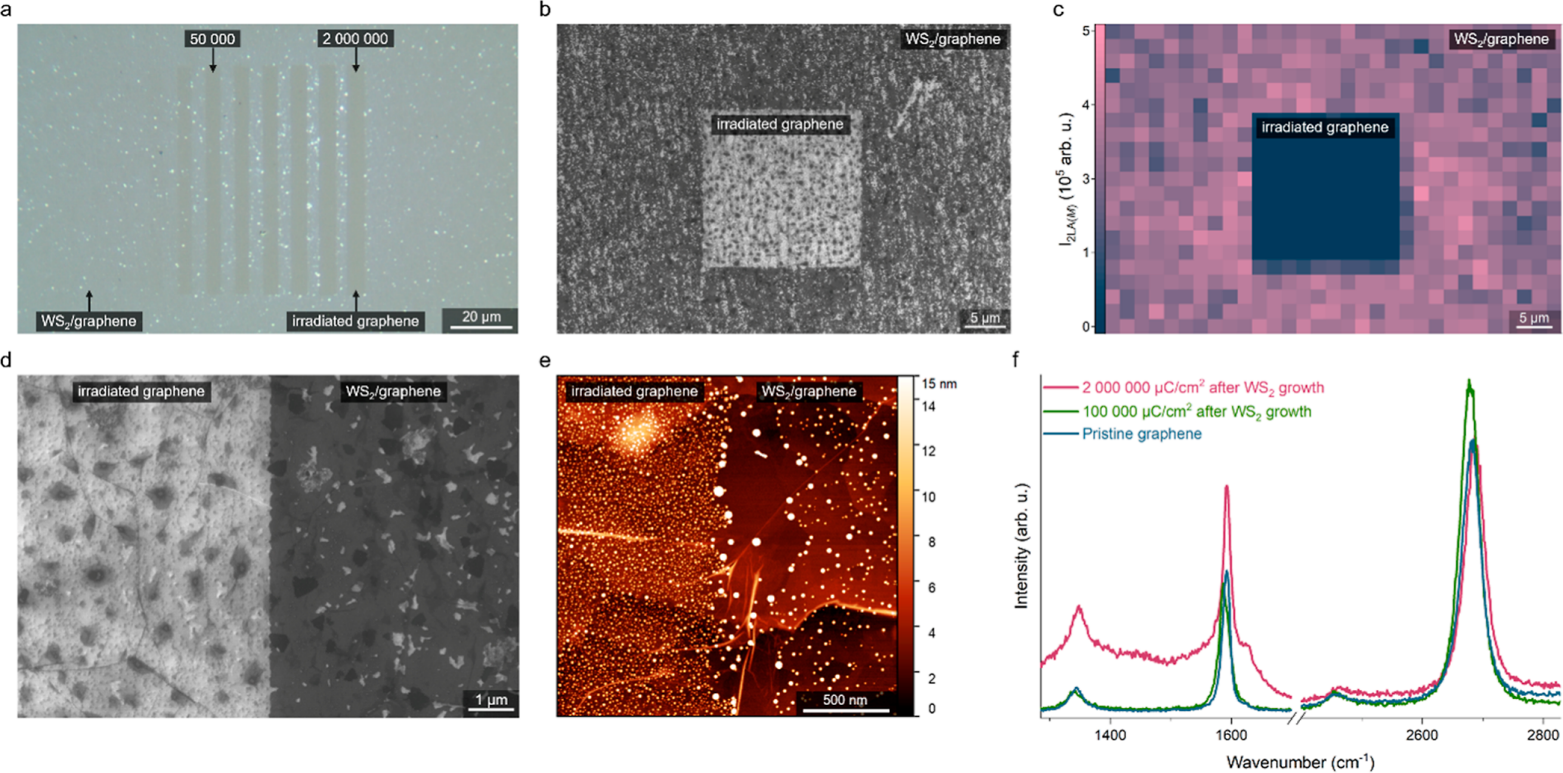
Selective negative growth of van der Waals heterostructures by EB irradiation on CVD graphene on sapphire. (a) Optical micrograph of WS_2_ synthesized in the negative growth mode on graphene. WS_2_ nucleated everywhere except the regions of graphene irradiated with doses higher than 50,000 μC/cm^2^. The electron doses are given in μC/cm^2^. (b) SEM image in in-lens contrast of WS_2_ synthesized in the negative growth mode on graphene. Similar to the optical micrograph, the irradiated 20 μm-square in the center of the image is free of WS_2_ growth. (c) Raman mapping of WS_2_ 2LA(*M*) peak. Confirming the optical and SEM imaging, WS_2_ did not nucleate on the irradiated graphene, while a nearly continuous layer is present on the non-irradiated areas. (d) SEM image of the sharp boundary between WS_2_ monolayer and irradiated graphene. (e) AFM image of the boundary region between non-irradiated graphene with WS_2_ monolayer and graphene irradiated with 1,000,000 μC/cm^2^. The irradiated areas are of higher thickness than pristine graphene with WS_2_. (f) Raman characterization of graphene after negative growth. While regions of up to 100,000 μC/cm^2^ healed to a pristine state, areas with higher doses remain highly disordered.

Besides the positive growth of van der Waals heterostructures, it is possible to achieve negative growth with EB irradiation on the graphene substrate, i.e., nucleation is prohibited in the irradiated areas. For this purpose, it is necessary to limit the time between EB irradiation and CVD growth to a maximum of 2 h, and simultaneously the graphene substrate cannot be exposed to ambient air for more than 20 min. As presented in Figure 3a,b, the negative growth effect is optically visible and further confirmed by Raman mapping of the WS_2_ 2LA(*M*) Raman peak (Figure 3c). Similar to the positive growth, the higher the electron dose, the more intense the effect. Notably, the effect is already fully achieved at 50,000 μC/cm^2^, and lower doses did not completely prevent nucleation.

The SEM micrograph (Figure 3d) shows that contrary to positive growth, the interface between irradiated and non-irradiated regions is exceptionally sharp—in fact, there is no interface region, and the boundary of the WS_2_ monolayer varies by less than 100 nm. An AFM image (Figure 3e) further confirms the sharp boundary and indicates that the WS_2_ growth region is less damaged than the irradiated graphene area. Interestingly, the irradiated graphene area is on average thicker by approx. 1.5 nm than the non-irradiated graphene with a WS_2_ monolayer. The height difference is explained by Raman studies (Figure 3f) that indicate the regions irradiated with the highest dose of 2,000,000 μC/cm^2^ did not heal and remained highly disordered. Still, the regions with a dose of 100,000 μC/cm^2^, so sufficient to completely prohibit TMDs growth, fully recovered to a pristine state (Figure 3f).

In addition to the synthesis experiments, we fabricated a photoconductor based on the negative WS_2_/graphene heterostructure. Among several applications, photodetectors and optical memories are the most promising devices based on graphene/TMD heterostructures.^34−36^ The selective growth method might be especially useful for fabricating photodetector arrays, mimicking the human eye.^37,38^ An SEM image and an *I*–*V* curve of our photoconductor with and without illumination are presented in Figure S12. The heterostructure exhibits negative photocurrent under illumination, which is in line with other studies.^36,39^ It proves the applicability of selective growth enabled by EB irradiation.

## 3 Discussion

**Figure 4:**
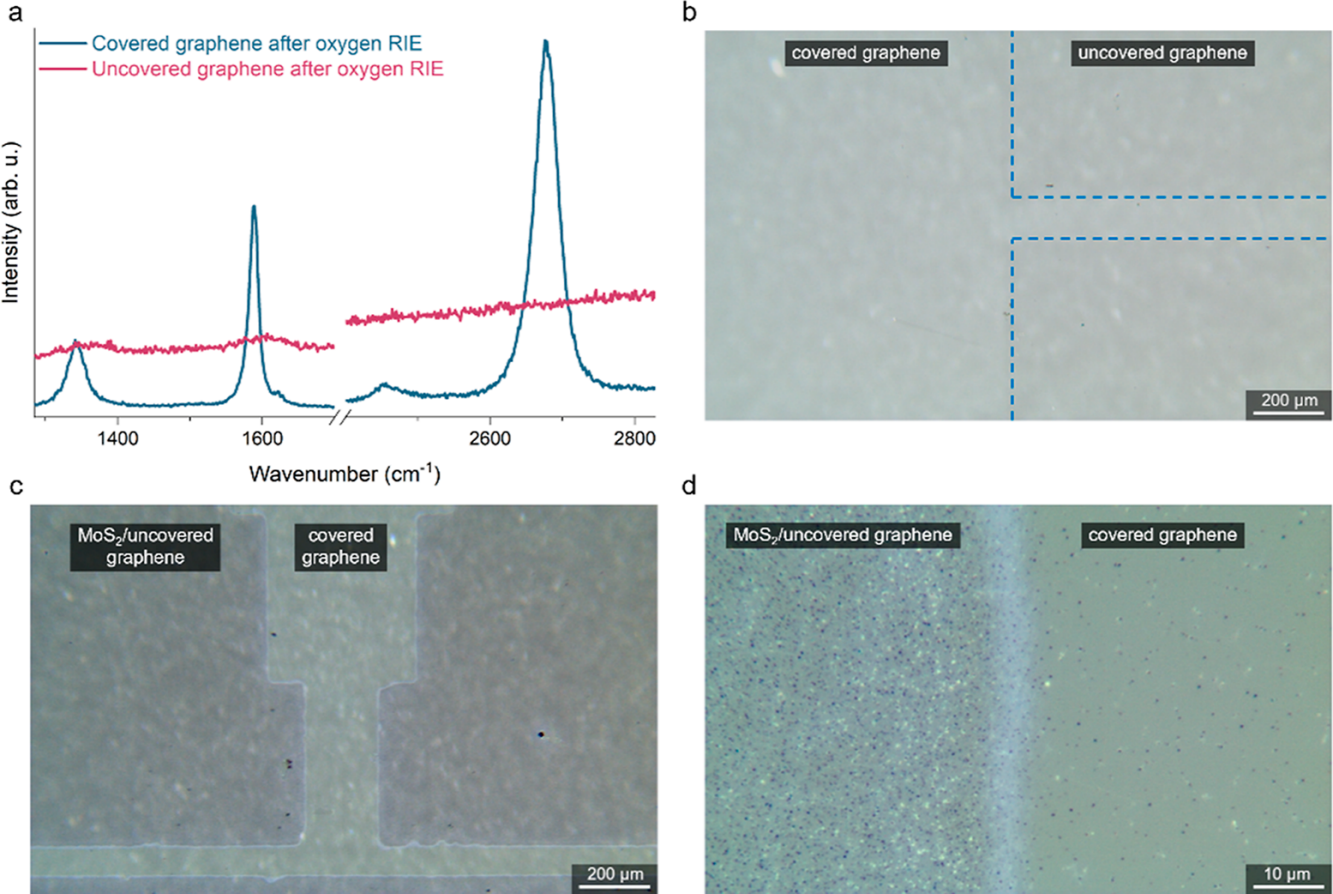
Selective positive growth of van der Waals heterostructures by oxygen RIE on CVD graphene on sapphire. (a) Raman spectra of the pristine and 1 s oxygen RIE graphene substrate. (b) Optical micrograph of the etched graphene substrate. The etched regions are visible as darker areas marked with dashed blue lines on the right side of the image. (c) RIE-etched graphene after MoS_2_ growth. MoS_2_ nucleated more readily in the irradiated areas. (d) The interface between the etched and unetched region is not as sharp as in the case of EB irradiation (Figure 2a).

To investigate the mechanisms governing selective growth, we aimed to determine whether mechanical degradation of graphene or hydrocarbon deposition during EB irradiation and aging is the cause of the observed effects. Hence, we degraded graphene by oxygen reactive ion etching (RIE) using a mechanical mask, excluding the impact of the hydrocarbon deposition. As presented in Figure 4a, the layer is extremely degraded even after 1 s of oxygen plasma treatment, resembling amorphous carbon more than graphene. Simultaneously, the optical image shows a faint difference between etched and covered regions (Figure 4b). After the growth of MoS_2_ on RIE-treated graphene, the effect of selective growth is clear, and MoS_2_ nucleated more easily on the etched regions (Figure 4c,d). However, the interface between etched and pristine graphene is not nearly as sharp an interface as in the EB method.

Therefore, the mechanism of selective growth by EB irradiation is at least partially based on a mechanical degradation of graphene and WS_2_ layers. It is explained by the fact that 2D materials nucleate more readily on the defects, or, more precisely, the dangling bonds—energetically favorable locations that serve as nucleation spots for 2D materials.^40^ Still, the mechanical degradation of the layer cannot explain the self-healing process observed in the positive and negative mechanisms. Thus, we suggest that during EB irradiation, a number of various carbon species are deposited on the surface of graphene. During the subsequent growth of MoS_2_ or WS_2_, these carbonaceous adsorbates serve as “repair kits” for the graphene layer, leading to the self-healing mechanism. Furthermore, in the case of positive growth, the graphene surface is exposed to ambient air and airborne contamination that can be adsorbed on the defective graphene. For this reason, the observed self-healing is more pronounced in the positive than negative growth. In addition, self-healing is not observed in the case of RIE-processed samples (Figure S13).

Nevertheless, the cause for the change from positive to negative growth mode is elusive. Despite the supplementary discussion on the 2D materials growth mechanism presented in Supporting Note 1, a clear conclusion cannot be made based only on thermodynamic and kinetic explanations. Still, a hypothesis for the negative growth cause can be based on electrostatic interaction. As presented in SEM and Raman characterization (Figure 1b,e), graphene and the underlying dielectric substrate are significantly charged. This effect can also be seen as a “halo” that is more intense in the regions irradiated with higher electron dose in the sample synthesized via MOCVD (Figure S14).

**Figure 5:**
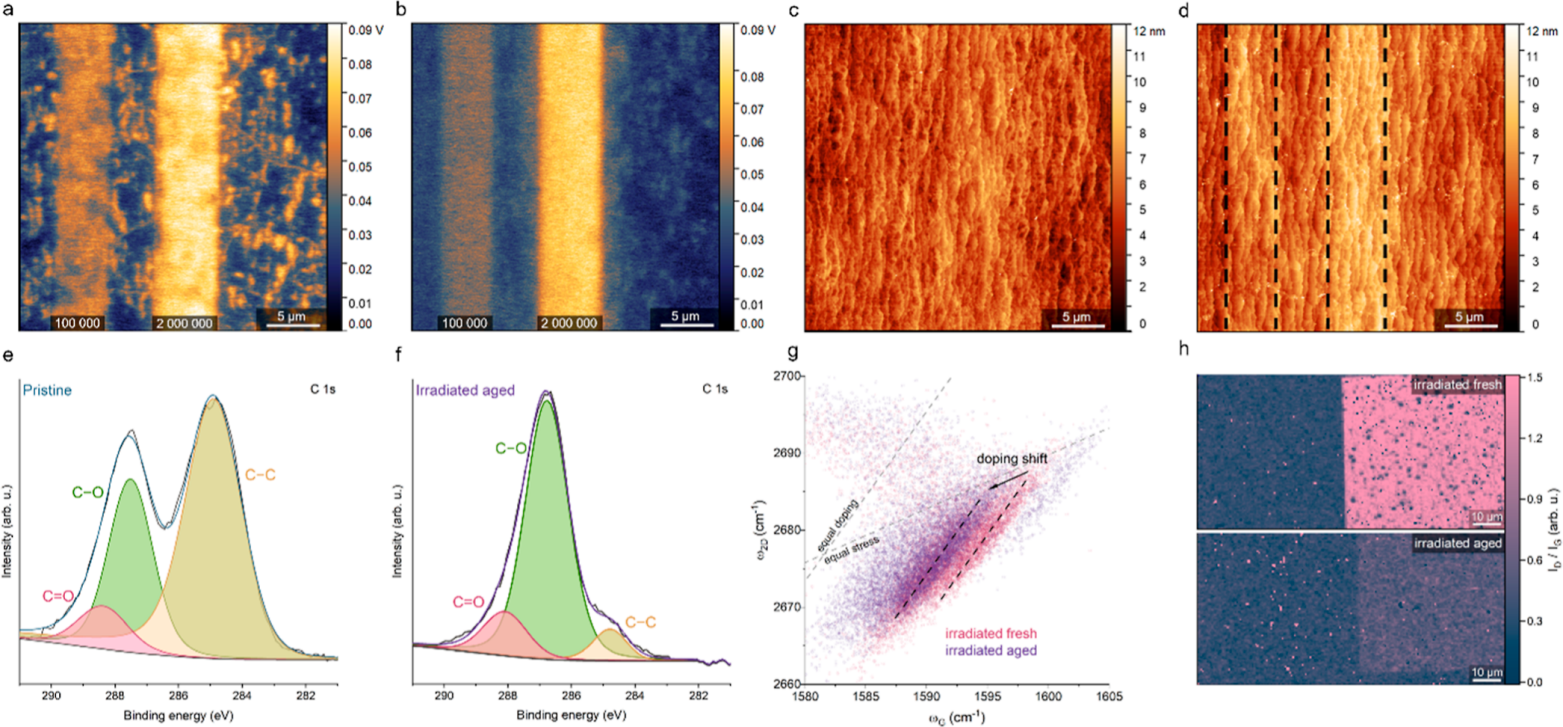
Characterization of fresh and aged irradiated graphene substrate. (a,b) Kelvin-probe force microscopy images of the irradiated graphene immediately after irradiation (a) and after aging for 66 h in ambient air at 70 °C (b). The electron doses at the bottom of the stripes are in μC/cm^2^. There is no difference in surface potential before and after aging, and the difference between graphene irradiated with 2,000,000 μC/cm^2^ and pristine graphene is maintained at approx. 60 mV. (c,d) Respective topography images of areas presented in (a,b). The thickness increase of the irradiated stripes (marked with dashed black lines) in the aged sample is approx. 1 and 3 nm for 100,000 and 2,000,000 μC/cm^2^, respectively. (e) XPS spectrum of C 1s of the pristine graphene region. (f) XPS spectrum of C 1s of the irradiated aged graphene region. (g) Raman strain-doping decomposition of graphene irradiated with 100,000 μC/cm^2^ before and after aging. The electron doping decreased after aging, while strain remained unchanged. (h) Graphene healing occurs even at 70 °C in ambient air, as shown by *I*_D_/*I*_G_ Raman mapping of areas irradiated with 100,000 μC/cm^2^.

To investigate the electrostatic cause of the negative growth, we characterized the fresh and aged irradiated graphene substrate. First, we performed Kelvin-probe force microscopy (KPFM) measurements of the graphene immediately after EB irradiation and after 66 h aging at 70 °C in ambient air (Figure 5a,b). The surface potential did not change after aging and is approx. 60 mV higher on graphene irradiated with 2,000,000 μC/cm^2^ dose than on pristine graphene. However, the topography of the irradiated regions differs significantly after the mild annealing (Figure 5c,d). After aging, the height of the irradiated regions increased by approx. 1 and 3 nm for 100,000 and 2,000,000 μC/cm^2^ doses, respectively. It proves that, indeed, carbon species attach during aging, further supporting our hypothesis of graphene self-healing.

High-resolution X-ray photoemission spectroscopy was conducted on the irradiated aged graphene substrate to further confirm the proposed selective growth mechanism. As shown in Figure 5e,f, the C 1s core-level spectrum of the modified and pristine regions of the sample shows a well-resolved peak at approx. 284.8 eV corresponding to graphene and peaks at approx. 286–289.6 eV that can be attributed to carbon oxides. The evolution of the C 1s spectrum after EB irradiation and atmospheric exposure indicates that the irradiated region was strongly modified by introducing defects into the graphene substrate.

We also conducted Raman spatial mapping of the irradiated graphene before and after aging. As presented in Figure 5g, the electron doping decreased after aging, recovering by a value similar to the increase in doping after irradiation (Figure 1f). Furthermore, we observed that even at very mild conditions of aging, the self-healing of graphene occurs, as confirmed by *I*_D_/*I*_G_ mapping (Figure 5h).

We argue that the charged surface decreased the adsorption of the precursors during the CVD growth. Although no reports show that charged particles are present in the CVD growth of TMDs,^41^ a very recent work showed a similar effect for CVD growth of graphene on copper.^42^ Furthermore, the effect of decreasing adsorption with negatively biased graphene and TMDs is well-known.^43^ Therefore, it is possible that the freshly-irradiated graphene with limited air exposure maintains its surface charge, which causes the limited adsorption of gaseous TMD precursors during the growth. However, when the time between EB irradiation and CVD growth is extended, the electrical charge is dissipated, and carbonaceous species can be easily adsorbed on the surface. As an effect of the long air exposure, we observe positive growth and extensive graphene self-healing.

In addition, we observed that one of the growth processes resulted in mixed negative and positive growth, and the positive and negative growth regions have different work functions (Figure S15). The regions with the three highest doses (500,000 to 2,000,000 μC/cm^2^) started to grow in positive growth mode, but lower doses were still negative. This suggests that there is a competition between defect-mediated and electrostatic-mediated growth.

To test the selective growth model further, we conducted simplified DFT studies considering a supercell with graphene and a W_3_S_6_ nucleus (Figure S16). The simulations indicate that the binding energy between graphene and WS_2_ is increased when vacancies are introduced to graphene. However, this energy is slightly decreased with the introduction of a negative charge (Table S1). These simulations align with the experimental results and indicate that defectiveness and electronic doping of the graphene substrate influence the nucleation of 2D materials.

## 4 Conclusions

In conclusion, we presented a method allowing selective growth of TMDs on graphene and WS_2_ substrates by EB irradiation. By irradiating 2D substrates with doses between 5000 and 2,000,000 μC/cm^2^, it is possible to achieve selective growth of MoS_2_ and WS_2_ by CVD and MOCVD. Notably, our results show that the optimal value of irradiation doses is between 10,000 and 100,000 μC/cm^2^. The growth mode can be manipulated by modifying the time between EB irradiation and growth and air exposure. If both periods are limited, the growth is negative, i.e., nucleation of TMDs is prohibited in the irradiated areas. In the other case, growth occurs in the EB-irradiated regions. Furthermore, our method allows for the self-healing of the graphene substrate. The self-healing is explained by the adsorption of carbon species during EB irradiation and exposure to ambient air, which provides carbon atoms necessary to reconstruct the perfect crystal lattice of graphene during the CVD growth at elevated temperatures.

The selective growth mechanism was investigated with multiple methods, including Raman mapping, KPFM, XPS, and DFT studies. Based on these results, we propose that the selective growth mechanism is based on a competition of three effects: mechanical degradation of the 2D substrate, adsorption of carbon species on substrate surfaces, and electrostatic interaction between the substrate and the precursor molecules. As this method is easily applicable via EB lithography and is suitable for a wide range of 2D materials, we suggest that it can be a critical step in simplifying the technological workflows of the production of 2D-materials-based nanoelectronics.

## 5 Methods

### 5.1 Substrate Preparation

CVD graphene transferred onto SiO_2_/Si, CVD graphene on sapphire, and CVD WS_2_ on CVD graphene on sapphire were used as substrates. Graphene transferred onto SiO_2_/Si was purchased from Graphenea. Polycrystalline CVD graphene was synthesized on 2 in. *c*-plane sapphire in an experimental close-couple showerhead AIXTRON reactor. The graphene growth was conducted at 1560 °C for 4 min with methane as a carbon precursor. The detailed graphene growth procedure was presented elsewhere.^44^ The WS_2_ substrate was synthesized on CVD graphene/sapphire according to the procedure presented in Section 5.2 below.

EB irradiation was conducted in Raith eLine plus EB lithography. Graphene on sapphire and graphene on SiO_2_/Si were irradiated with doses ranging from 1000 to 2,000,000 μC/cm^2^ in an array of different shapes presented in Figure S1. The electron beam was focused to 2 nm, and the irradiation was done in 200 × 200 nm steps. For positive growth, the as-irradiated samples were kept in the air at room temperature for at least 24 h, and the best results were achieved by aging the samples on a hot plate at 70 °C for 66 h. For the negative growth, the substrates were transferred from EB lithography to the CVD reactor in under 1 h. These samples were vacuum-packed for transportation, so the total air exposure was limited to approx. 10 min. The WS_2_/graphene substrate for the selective growth of MoS_2_ was kept in the SEM chamber for 16 h under a vacuum of 10^–6^ mbar and then investigated using standard parameters described in Section 5.3 below.

### 5.2 Synthesis of MoS_2_ and WS_2_

CVD growth of MoS_2_ and WS_2_ was conducted in a 2 in. tube furnace at 770 and 900 °C, respectively. As precursors, sublimed sulfur (Chempur, pure p.a.) and MoO_3_ (Alfa Aesar, 99.95%) or WO_3_ (Alfa Aesar, 99.8%) were used with a small addition (2–10 mg) of NaCl (Carl Roth, 99.8%). The growths were conducted for 15 min in an Ar atmosphere. The detailed MoS_2_ and WS_2_ growth procedures were presented elsewhere.^45,46^

MOCVD growth of MoS_2_ was conducted in a 4 in. tube furnace using molybdenum hexacarbonyl and diethyl sulfide as precursors under a hydrogen and argon atmosphere. The growth was conducted at 870 °C for 60 min. The detailed MoS_2_ growth procedure was presented elsewhere.^47^

### 5.3 Characterization

The morphology of the samples was investigated using a Raith eLine plus scanning electron microscope with in-lens and secondary electron detectors. Typically, the samples were characterized with 1 and 10 kV accelerating voltage with 6.2 mm working distance and 7 μm aperture.

A Bruker Dimension Icon atomic force microscope allowed investigation of the samples’ topography in tapping mode using supersharp tips (nominal radius 1 nm) and surface potential in Kelvin-probe mode using Pt–Ir coated tips with 25 nm nominal radius.

Chemical composition and photoluminescence were measured using a Renishaw inVia Qontor Raman spectrometer. A 532 nm laser with 12.5 mW and ×100 objective was used for these measurements. The Raman spatial maps were typically measured using 500 and 2000 nm steps.

The structural properties of the WS_2_/graphene interface have been investigated with an FEI-Titan 80-300 transmission electron microscope operating at 300 kV, equipped with an image corrector. Before sample preparation, needed for TEM purposes, the investigated material was covered with an amorphous carbon protective layer of approximately 5 nm. Subsequently, an FEI-Helios Nanolab 600 FIB was used to prepare the WS_2_/graphene/sapphire interface cross-section specimen by a focused ion beam (FIB) equipped with an OmniProbe nanomanipulator and platinum gas injection system (GIS). The standard polycrystalline platinum layer was deposited on the specimen to protect the material from damage during FIB lamella cutting out. The elemental composition determination was performed by EDX using an EDAX 30 mm^2^ Si(Li) detector.

XPS measurements were performed at room temperature in a UHV Multiprobe P (Scienta-Omicron) system with a base pressure of 5 × 10^–10^ mbar. A hemispherical energy analyzer Phoibos 150 (SPECS) with a 2D-CCD detector and the DAR 400 X-ray source with Mg Kα (1253.64 eV) non-monochromatic radiation was used. The XPS spectra were analyzed with the CasaXPS software. The XPS data analysis involved Shirley background subtraction and curve-fitting (mixed Gaussian–Lorentzian function with 85% of Gaussian for carbon oxides and asymmetric Doniach-Sunjic line shape for graphene).

### 5.4 Theoretical Modeling

We performed theoretical simulations using the DFT method as implemented in the Quantum ESPRESSO v.7.0 software.^48−50^ To study the binding energies between the as-grown 9-atom WS_2_ clusters (W_3_S_6_) and graphene layers (pristine, defected, or charged), we considered 4 × 4 graphene supercells (31–32 carbon atoms) with the adsorbed W_3_S_6_ cluster at one side (Figure S16). The details of the modeling are presented in Supporting Note 2.

### 5.5 Device Fabrication

Using Raith eLine plus EB lithography, followed by a lift-off process, we fabricated 5 nm Cr/100 nm Au contacts to the vdWHS layers. The electrical measurements have been done with National Instruments DAQ USB-6366 and current preamplifier DL Instruments 1211. White halogen light provided scattered illumination.

### 5.6 Structural Modification of the Device

Before electrical measurements, the devices were treated with surface cleaning and sensitizing plasma treatment. The plasma was provided by a Diener electronic Zepto plasma cleaner with the gas mixture of argon and oxygen of equal proportions (15 SCCM gas flow, 3 s duration time). The treatment cleaned the surface of the aged sample and enhanced the effect of photoconductivity observed in WS_2_.

## Supporting Information Available

Typical irradiation matrix and SEM image of the irradiated graphene; SEM images of irradiated features in in-lens and SE imaging; SEM image of the accuracy of the EB irradiation; exemplary Raman spectra of the graphene irradiated with all investigated doses; detailed Raman mapping of the irradiated graphene; optical and SEM images of the selective growth on MoS_2_ on WS_2_; AFM of the irradiation matrix with WS_2_ nuclei; HRTEM and EDX results of WS_2_/graphene heterostructure; PL mapping of large WS_2_ flake synthesized in positive growth mode; optical image and Raman mapping showing the self-healing of graphene in positive growth mode; SEM and AFM images of WS_2_ synthesized in the positive growth mode; SEM image and electrical characterization of photoconductor; Raman spectra of RIE-etched graphene after growth process; SEM image of MOCVD-grown MoS_2_; optical and SEM images of the mixed negative-positive growth process; and images of the simulated WS_2_/graphene models (PDF)
