# Supplementary material for: Selective Growth of van der Waals Heterostructures Enabled by Electron-Beam Irradiation
Source: ACS Appl Mater Interfaces. 2023 Jul 7;15(28):33838–47. doi: 10.1021/acsami.3c02892 (PMC10360032; doi:10.1021/acsami.3c02892)
Supplement: Supplementary file 1 — am3c02892_si_001.pdf [file am3c02892_si_001.pdf]

Supporting information to:

Selective growth of van der Waals heterostructures enabled by electron-beam irradiation

Jakub Sitek[1,2†], Karolina Czerniak-Łosiewicz[1], Arkadiusz P. Gertych[1], Małgorzata Giza[1], Paweł Dąbrowski[3], Maciej Rogala[3], Konrad Wilczyński[1], Anna Kaleta[4], Sławomir Kret[4], Ben R. Conran[5], Xiaochen Wang[5], Clifford McAleese[5], Michał Macha[6, ‡], Aleksandra Radenović[6], Mariusz Zdrojek[1], Iwona Pasternak[1], Włodek Strupiński[1]*

[1] Faculty of Physics, Warsaw University of Technology, Koszykowa 75, 00-662 Warsaw, Poland

[2] CENTERA Laboratory, Institute for High Pressure Physics, Polish Academy of Sciences, Sokołowska 29, 01-142 Warsaw, Poland

[3] Faculty of Physics and Applied Informatics, University of Łódź, Pomorska 149/153, 90-236 Łódź, Poland

[4] Institute of Physics, Polish Academy of Sciences, Al. Lotników 32/46, 02-668 Warsaw, Poland

[5] AIXTRON Ltd, Buckingway Business Park, Anderson Road, Swavesey, Cambridge, CB24 4FQ, United Kingdom

[6] Laboratory of Nanoscale Biology, Swiss Federal Institute of Technology Lausanne, Station 17, CH-015 Lausanne, Switzerland

† Current address: Center for Multidimensional Carbon Materials (CMCM), Institute for Basic Science (IBS), Unist-gil 50, Ulsan 44919, Republic of Korea

‡ Current address: European Organization for Nuclear Research (CERN), Esplanade des Particules 1, 1211 Geneve 23, Switzerland

*email: jakub.sitek@pw.edu.pl

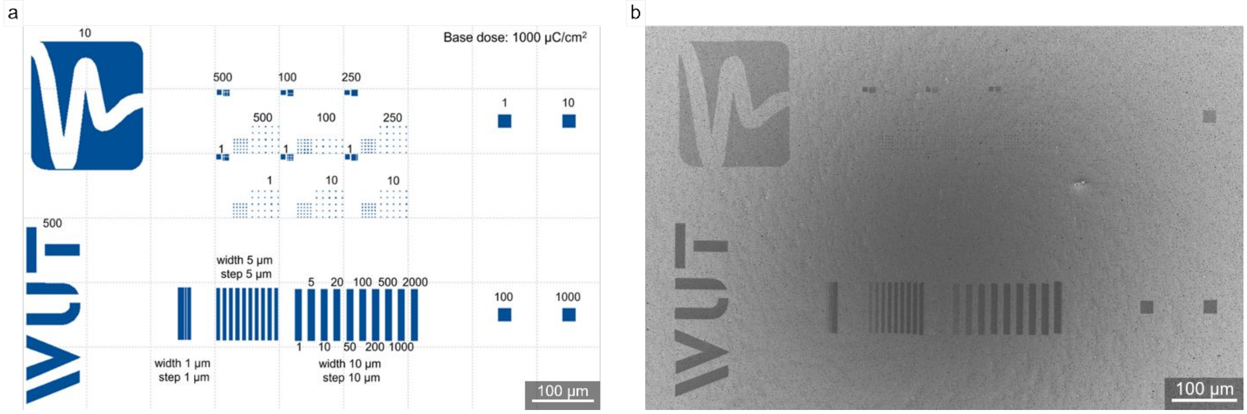

Figure S1. Typical irradiation matrix. (a)The irradiation matrix with the applied electron doses. The base irradiation dose is 1000 µC/cm^2, and the dose multiplayer is given on the scheme next to each feature. (b) Resultant irradiation pattern visible in SEM in-lens detector.

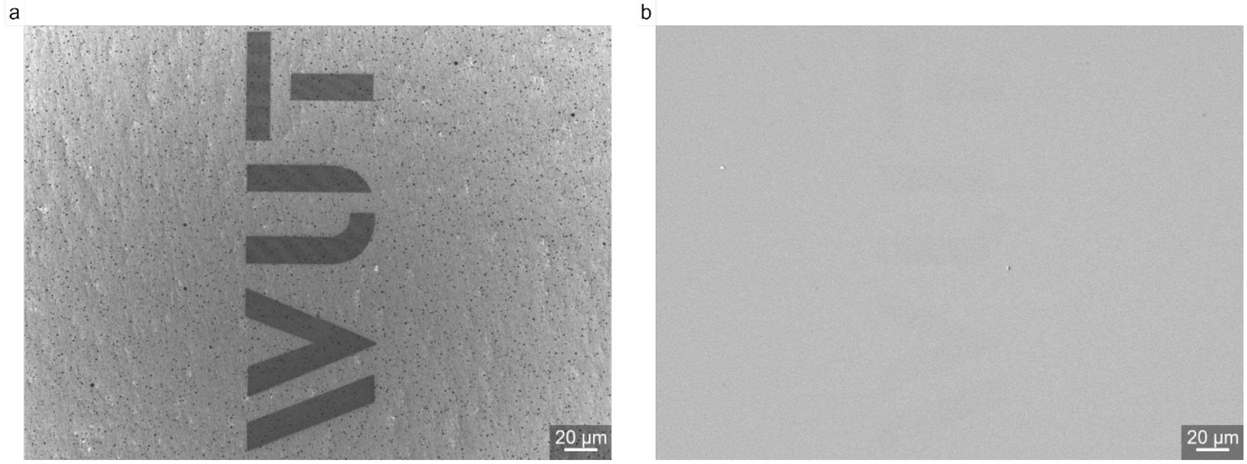

Figure S2. The "WUT" feature irradiated on graphene with a dose of 500 000 µC/cm^2 is visible in the in-lens contrast (a), but hardly distinguishable in secondary electrons contrast (b).

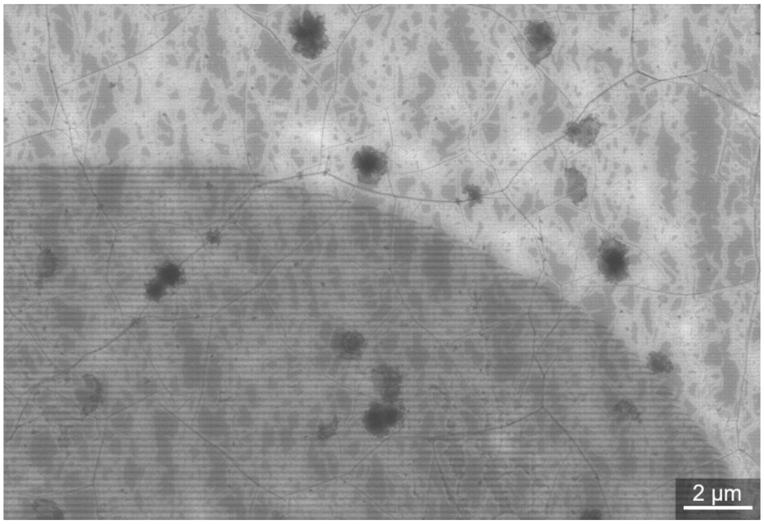

Figure S3. SEM in-lens image of the edge of the top left-most feature, “logo,” presented in Figure S1 showing exceptional precision of the electron-beam irradiation.

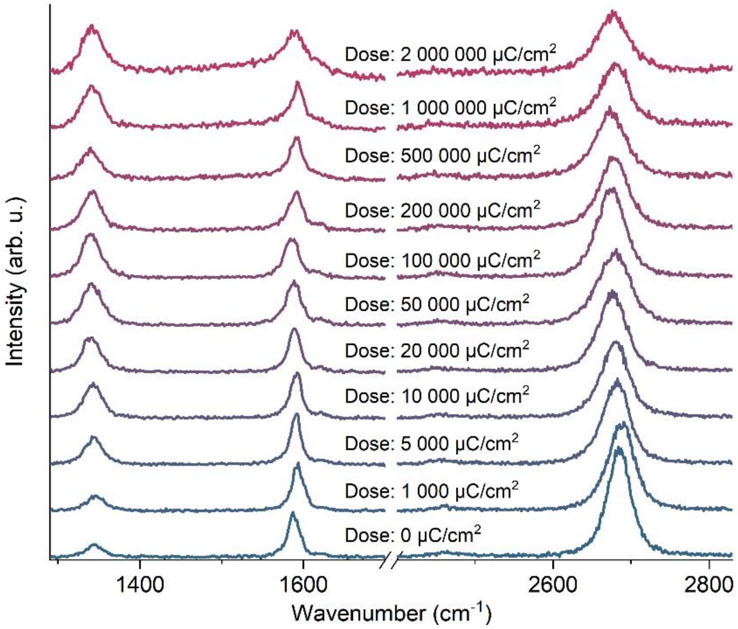

Figure S4. Exemplary Raman spectra of graphene irradiated with all investigated doses.

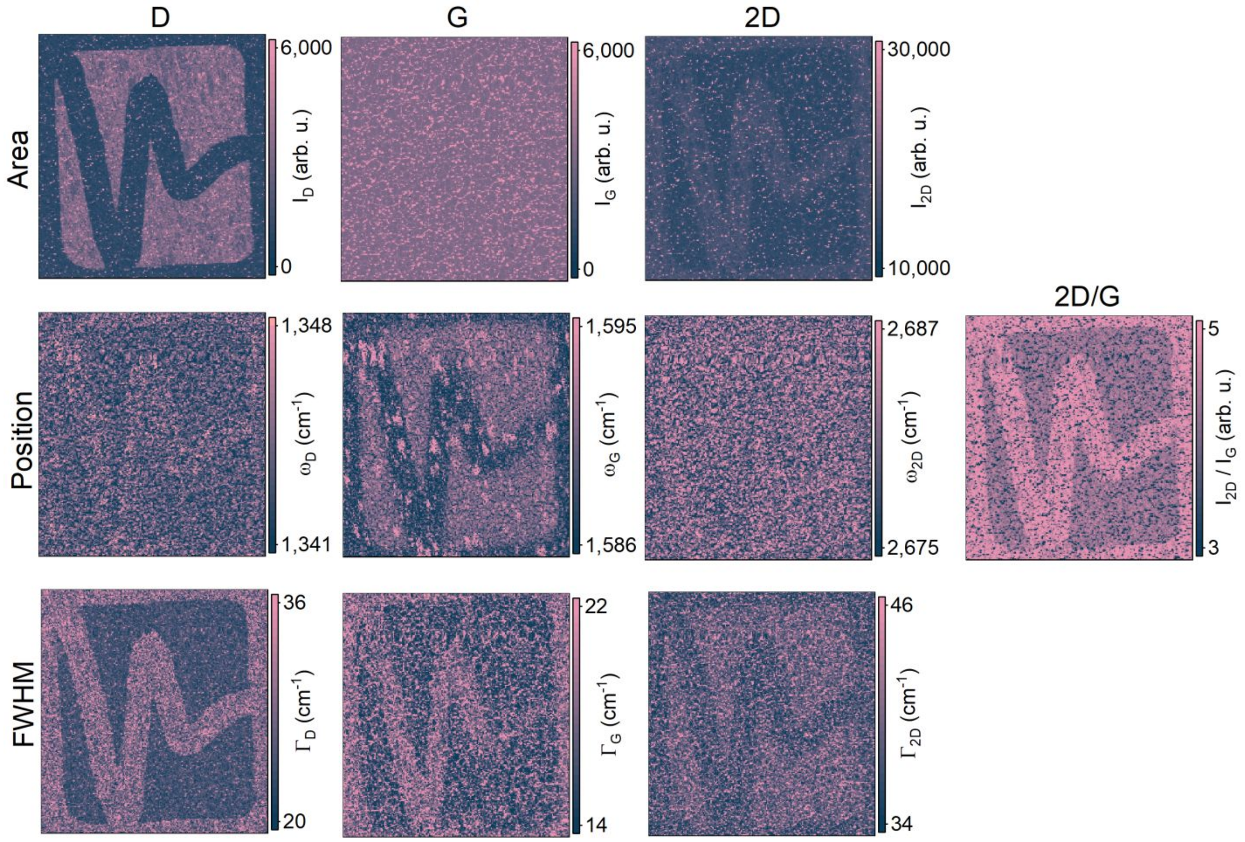

Figure S5. A detailed Raman mapping of the irradiated graphene substrate. The integrated area, position, and full width at half maximum of D, G, and 2D Raman peaks, and D/G and 2D/G integrated area ratios of graphene irradiated with 100 000 μC/cm^2 are presented.

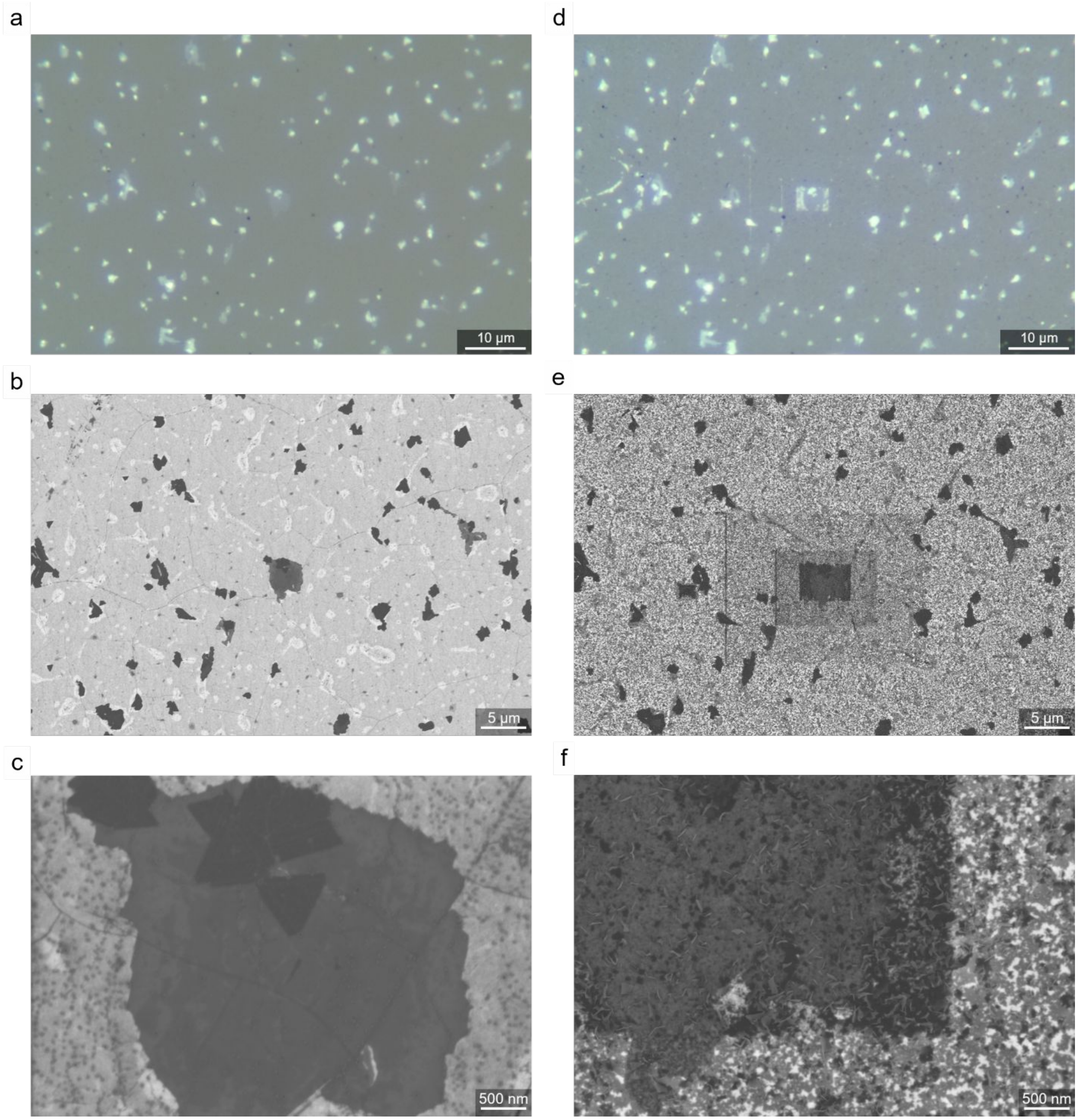

Figure S6. Selective positive growth of MoS_2 on WS_2/graphene substrate by SEM imaging. (a-c) A single area of the WS_2/graphene heterostructure characterized by standard SEM imaging. The substrate was kept in the SEM chamber in a vacuum for 16 hours prior to SEM characterization.

(d-f) The same area after the growth of MoS_2 on top. The areas previously investigated with SEM have higher coverage of MoS_2 than non-irradiated regions.

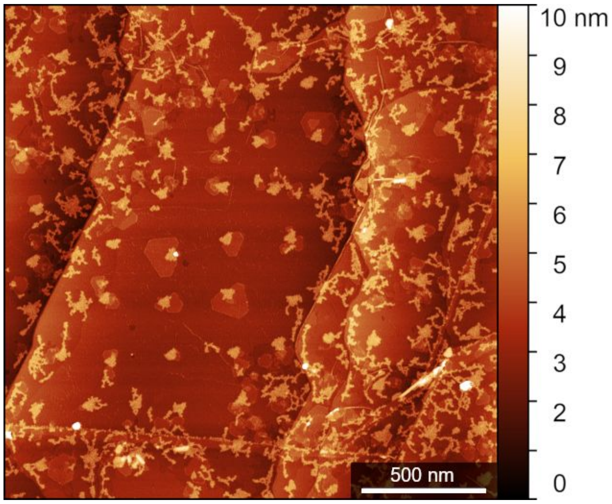

Figure S7. AFM image of the irradiation matrix, showing preferential nucleation at the irradiation spots.

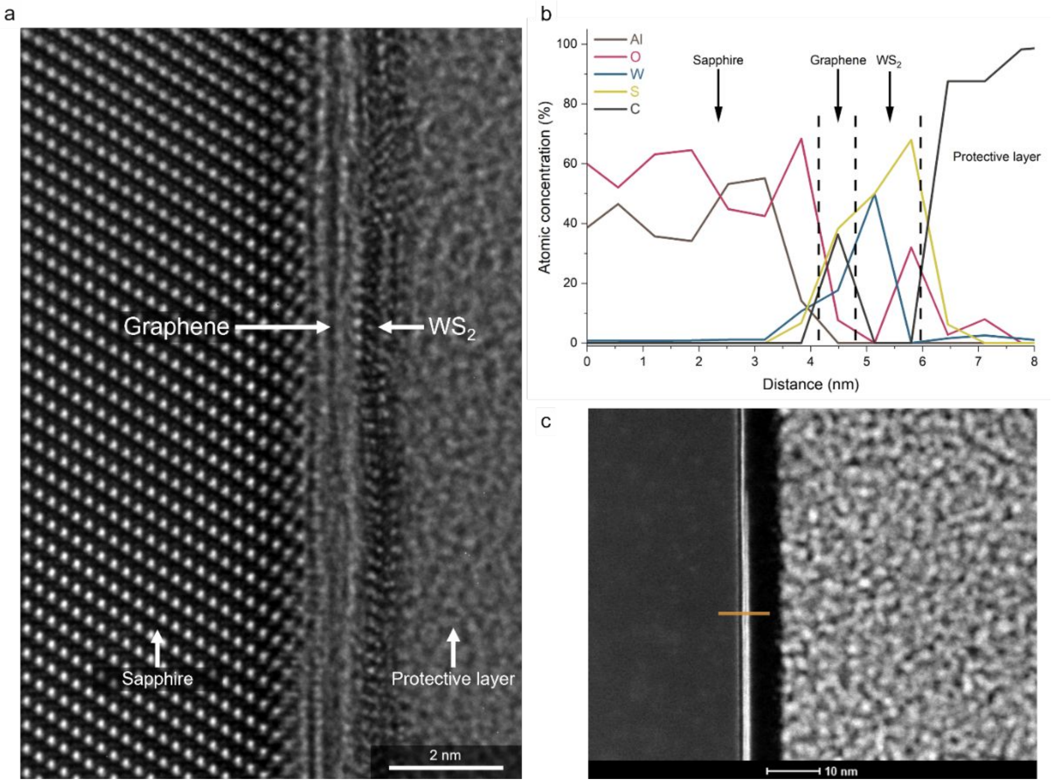

Figure S8. TEM studies of the synthesized WS_2/graphene heterostructure. (a) High-resolution TEM (HRTEM) image of the monolayer graphene/monolayer WS_2 heterostructure. The measured distances between substrate and graphene (3.3 Å) and graphene and WS_2 layer (6.2 Å) correspond to relevant van der Waals distances, which indicates that the interface is free of impurities. (b) EDX spectra of the heterostructure. Graphene and bilayer WS_2 can be easily distinguished in the spectrum. (c) Scanning TEM (STEM) image presenting the region where the EDX spectrum was collected. The scan line is marked with an orange line. The spectrum was collected on bilayer WS_2 to achieve a better signal-to-noise ratio. WS_2 bilayer is observed with the brightest intensity in

STEM Z-contrast imaging due to very high Z-number of tungsten. For the same reason, the first protective carbon layer is dark (low Z-number). The thick layer visible on the right side of the image corresponds to the second protective layer (used during FIB preparation) made of polycrystalline platinum.

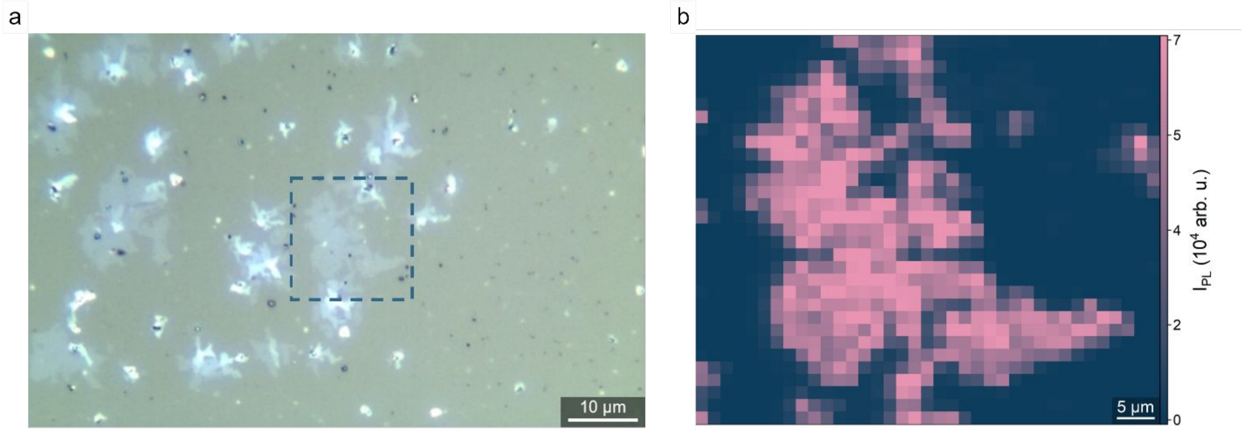

Figure S9. Uniformity of WS_2 photoluminescence after positive growth. (a) Optical micrograph of large WS_2 flakes synthesized in the “logo” region. (b) Mapping of WS_2 PL intensity of the flake shown in (a) with a blue rectangle, indicating a high and uniform PL emission.

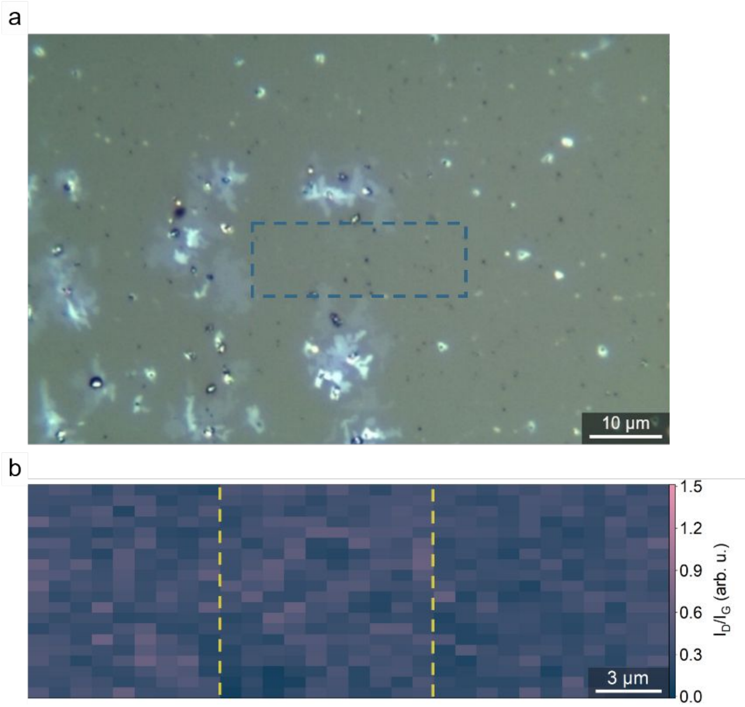

Figure S10. Graphene self-healing after positive growth of WS_2. (a) Optical micrograph of WS_2 flakes synthesized in the "stripes" region. (b) Mapping of graphene I_D/I_G ratio of the region shown in (a) with a blue dashed rectangle, indicating fully healed graphene after the growth process. The irradiated area is marked with yellow dashed lines. The translational steps in the *x* and *y* directions were 1 and 0.5 µm, respectively.

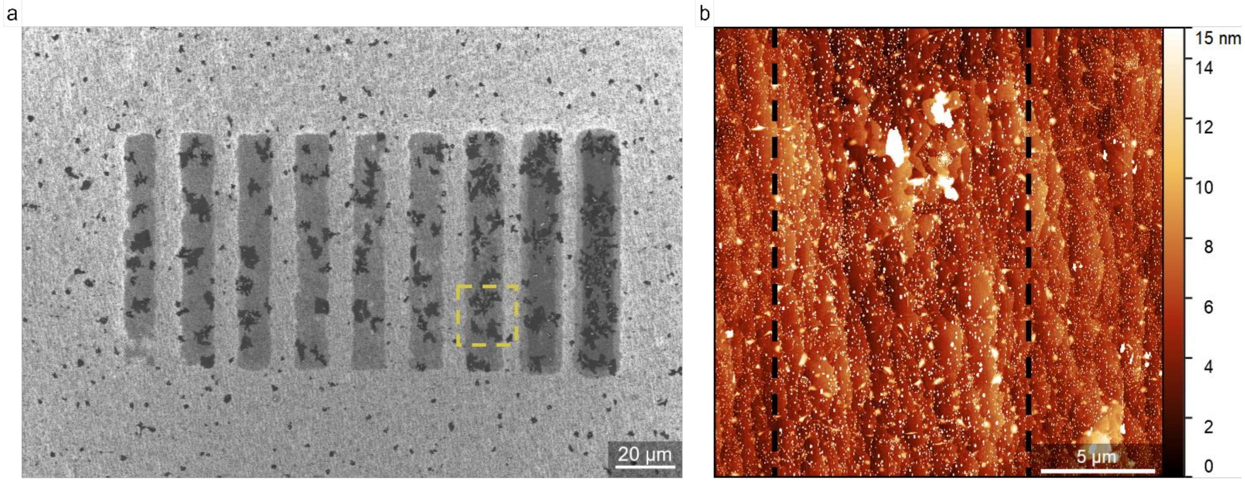

Figure S11. Graphene substrate after positive growth of WS_2. (a) In-lens SEM image of the stripes after positive growth. (b) AFM image of the regions shown in (a) with a yellow dashed square. There is no substantial difference in roughness between the non-irradiated area and the irradiated stripe (marked with black dashed lines).

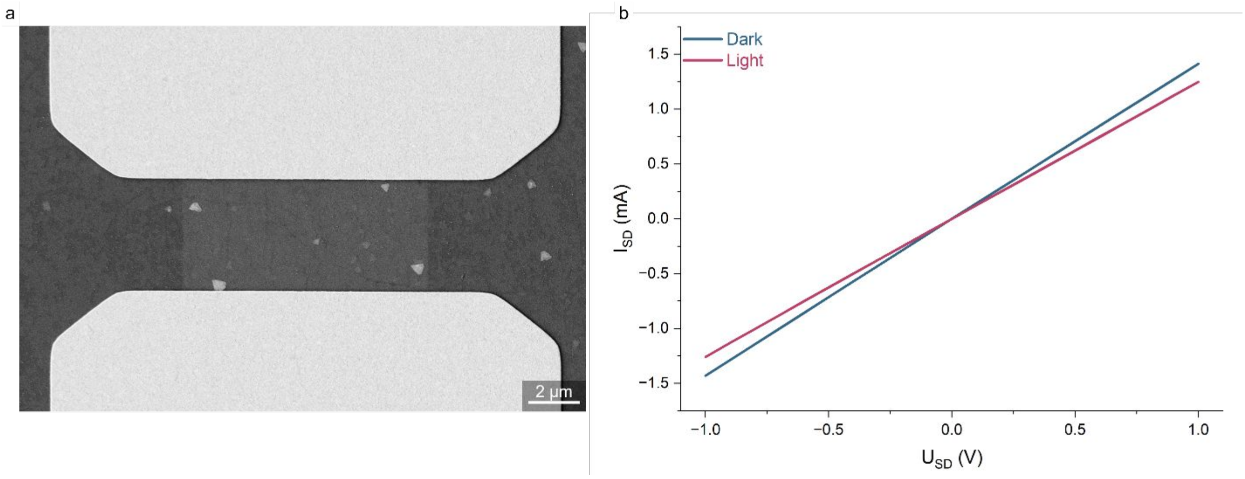

Figure S12. The fabricated photoconductor. (a) SEM image of the device. (b) I-V curve of the device showing a decrease in photocurrent under illumination.

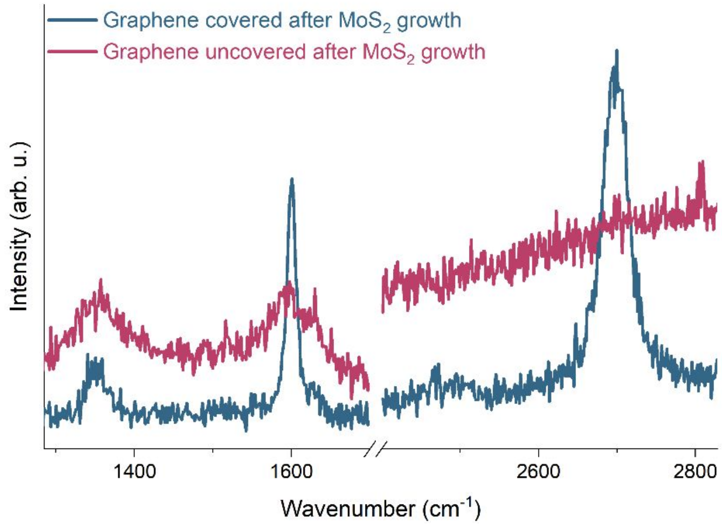

Figure S13. Raman characterization of the graphene etched with oxygen RIE after MoS_2 growth. The areas uncovered with a mechanical mask do not show self-healing.

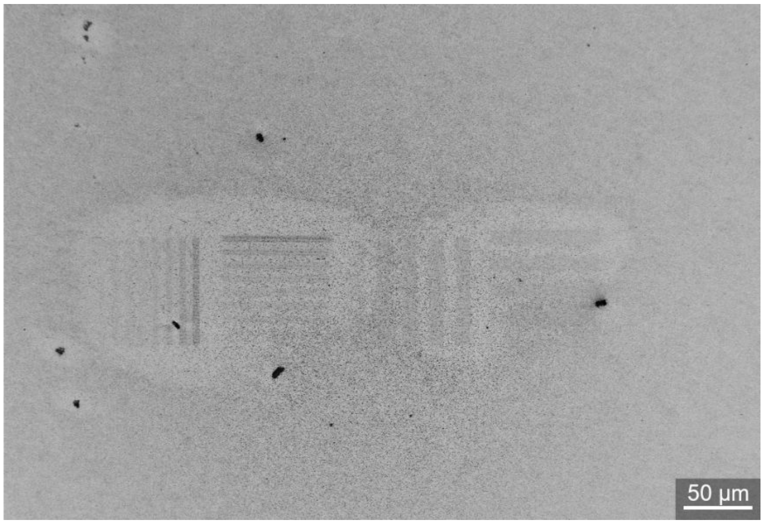

Figure S14. SEM in-lens image of MoS_2 grown by MOCVD on graphene. The "halo" around regions with higher irradiation doses is more pronounced than in the lower-dosed regions.

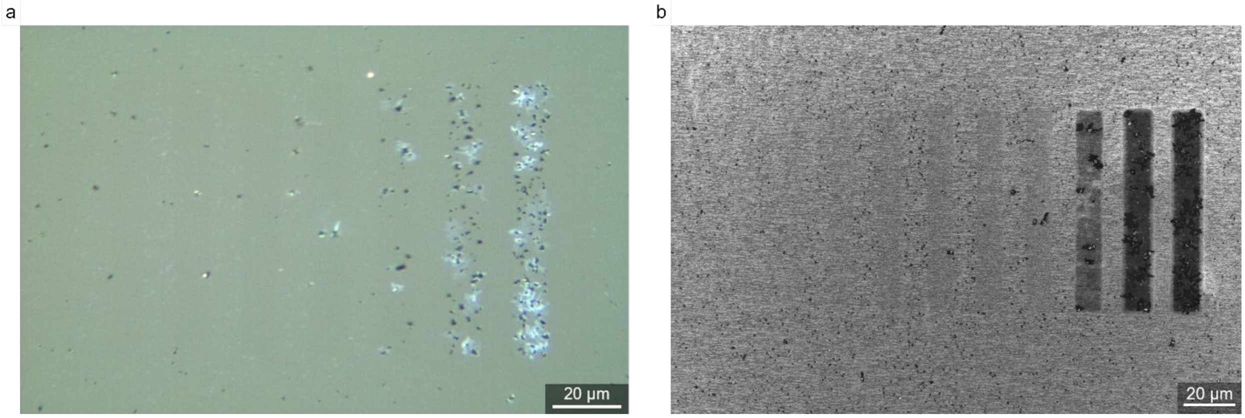

Figure S15. Mixed growth by EB irradiation. (a) Optical micrograph of mixed growth, with the three most-right stripes exhibiting positive growth, while others are negative. (b) SEM in-lens image of the same areas as in (a), showing much higher contrast in the positive growth region.

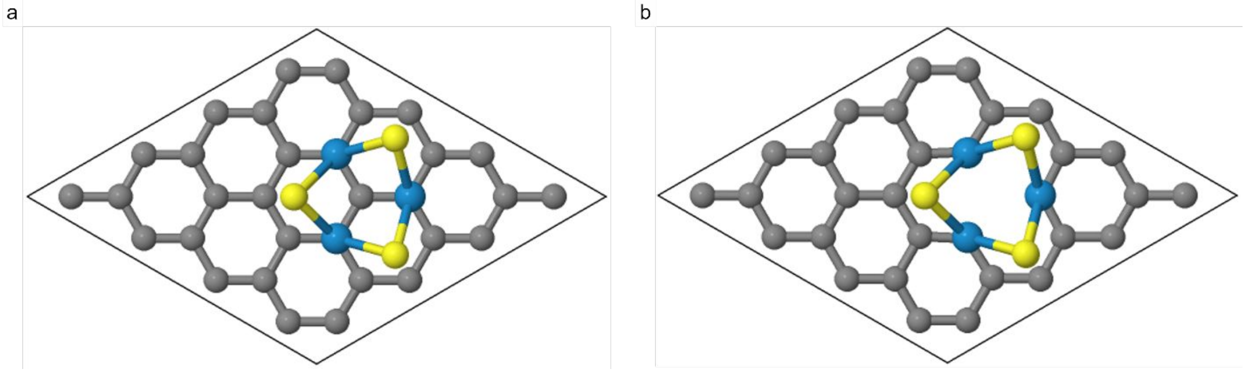

Figure S16. Images of models used in DFT simulations. (a) Pristine graphene with a W_3S_6 nucleus. (b) Defected graphene with a W_3S_6 nucleus.

Table S1. The calculated binding energy for different types of WS_2/graphene heterostructures.

**Type of heterostructure**	**Binding energy for a supercell (eV)**

Pristine graphene + WS_2	0.795
Defected graphene + WS_2	0.874
Negatively charged (5×10^{12} *e*/cm^2), defected graphene + WS_2	0.862

Supporting Note 1

Two main factors must be discussed to describe the growth of 2D materials: thermodynamics and kinetics.[1] Thermodynamics encompasses factors like temperature, pressure, type and flux of precursors, or the substrate-dependent energy of the layer formation. Kinetics discusses phenomena like mass transport, boundary layer, diffusion through the boundary layer, and surface diffusion.

Based solely on these factors, it is impossible to explain the negative growth. Factors like temperature, pressure, gas flows, precursors, and mass transport are identical for both growth modes; hence, the substrate is the critical factor, as discussed in our previous publications.[2–4] Substrate can impact growth in several ways; however, nucleation and surface diffusion are the most important factors.

Nucleation of 2D materials occurs more readily on the defects due to the lowered binding energy.[5] In this study, we induce defects in graphene, as confirmed by Raman spectroscopy (Figure 1 c). Therefore, we expect the nucleation to be higher on the more defected sites, which we observe in the positive growth (Figure 2). Still, we expect some defects to be passivated during air exposure, but a significant number of dangling bonds will be preserved. However, in the case of

negative growth, the degraded graphene layer has a limited time to be partially passivated during air exposure. Thus, it should result in even higher nucleation of TMDs, which is not what we observe.

The substrate surface also impacts the kinetics of the reaction, especially surface diffusion. Surface diffusion is generally hindered at surfaces with dangling bonds and is more unrestricted on 2D materials.[4,6,7] In the case of positive growth, it is possible that the reduced mobility of adsorbates results in larger domains, as 2D materials typically form larger domains on 3D substrates like SiO_2 and sapphire than on other 2D materials, including graphene.[3,8] However, it cannot explain why the nucleation does not occur in the negative growth mode – it is expected that the domains should be even larger than in the positive growth mode. In conclusion, we cannot explain the negative growth mode based only on thermodynamic and kinetic descriptors of the growth.

Supporting Note 2

For DFT studies, we used supercells with the height of 30 Å (which provided ~23.5 Å of vacuum separation). During the structures' relaxation, we converged all components of all atomic forces

below 70 meV/Å and the stress tensor components below 0.2 kbar. The binding energies were calculated using the formula (Equation (1)):

$$\Delta E_{\mathrm{tot}} = E(\mathrm{Gr} + W_3\mathrm{S}_6) - E(\mathrm{Gr}) - E(W_3\mathrm{S}_6), \#(1)$$

where $E(\mathrm{Gr} + \mathrm{W_3S_6})$ is the energy of relaxed graphene with the adsorbed W_3S_6 cluster, and E (Gr), $E(\mathrm{W_3S_6})$ are energies of graphene (with a defect or a charge, if applicable) and W_3S_6 cluster, respectively. The extra charge (corresponding to 5×10^{12} *e*/cm, in line with the Raman results) was realized by adding a fraction ~0.043 of an electron in the supercell. To account for the adhesion energy only, rather than the total energy difference between the thermodynamically stable systems, we evaluated the energies $E(\mathrm{Gr})$ and $E(\mathrm{W_3S_6})$ using the same positions of C, W, and S atoms. The illustrations of the modeled systems (Figure S16) were created using the Jmol 14.6.4 software.[9]

We performed all theoretical simulations using Quantum ESPRESSO v.7.0 software.[10–12] All atoms were represented using scalar relativistic pseudopotentials available at the Pseudo-Dojo database[13] (type ONCVPSP v0.4.1,[14] originally adapted for the PBE-GGA functional).[15] To properly represent the van der Waals interactions,[16,17] we used the vdW-D2[18] nonlocal exchange-correlation functional as implemented by the Thonhauser group.[19–21] We used plane-wave basis sets with cutoffs of 90 Ry and 270 Ry for wavefunctions and electronic density, respectively. The Brillouin zone of each supercell was sampled using the Monkhorst–Pack $\boldsymbol{k}$-point 5×5×1 (Γ-centered) mesh. We smeared the electronic levels' occupations using the Fermi-Dirac broadening corresponding to the temperature of 300 K.[22]

REFERENCES

(1) Herman, M. A.; Richter, W.; Sitter, H. Epitaxy: Physical Principles and Technical Implementation; Springer Series in MATERIALS SCIENCE; Springer Berlin Heidelberg: Berlin, Heidelberg, 2004. https://doi.org/10.1007/978-3-662-07064-2.

(2) Sitek, J.; Pasternak, I.; Grzonka, J.; Sobieski, J.; Judek, J.; Dabrowski, P.; Zdrojek, M.; Strupinski, W. Impact of Germanium Substrate Orientation on Morphological and Structural Properties of Graphene Grown by CVD Method. *Appl. Surf. Sci.* **2020**, *499*, 143913. https://doi.org/10.1016/j.apsusc.2019.143913.

(3) Sitek, J.; Plocharski, J.; Pasternak, I.; Gertych, A. P.; McAleese, C.; Conran, B. R.; Zdrojek, M.; Strupinski, W. Substrate-Induced Variances in Morphological and Structural Properties of MoS_2 Grown by Chemical Vapor Deposition on Epitaxial Graphene and SiO_2. *ACS Appl. Mater. Interfaces* **2020**, *12* (40), 45101–45110. https://doi.org/10.1021/acsami.0c06173.

(4) Sitek, J.; Pasternak, I.; Czerniak-Łosiewicz, K.; Świniarski, M.; Michałowski, P. P.; McAleese, C.; Wang, X.; Conran, B. R.; Wilczyński, K.; Macha, M.; Radenović, A.; Zdrojek, M.;

Strupiński, W. Three-Step, Transfer-Free Growth of MoS_2/WS_2/Graphene Vertical van der Waals Heterostructure. *2D Mater.* **2022**, *9* (2), 025030. https://doi.org/10.1088/2053-1583/ac5f6d.

(5) Wan, Y.; Fu, J. H.; Chuu, C. P.; Tung, V.; Shi, Y.; Li, L. J. Wafer-Scale Single-Orientation 2D Layers by Atomic Edge-Guided Epitaxial Growth. *Chem. Soc. Rev.* **2022**, *51* (3), 803–811. https://doi.org/10.1039/d1cs00264c.

(6) Oura, K.; Katayama, M.; Zotov, A. V.; Lifshits, V. G.; Saranin, A. A. Surface Science: An Introduction; Advanced Texts in Physics; Springer Berlin Heidelberg: Berlin, Heidelberg, 2003. https://doi.org/10.1007/978-3-662-05179-5.

(7) Sun, C.; Bai, B. Gas Diffusion on Graphene Surfaces. *Phys. Chem. Chem. Phys.* **2017**, *19* (5), 3894–3902. https://doi.org/10.1039/c6cp06267a.

(8) Eichfeld, S. M.; Hossain, L.; Lin, Y.-C. C.; Piasecki, A. F.; Kupp, B.; Birdwell, A. G.; Burke, R. A.; Lu, N.; Peng, X.; Li, J.; Azcatl, A.; McDonnell, S.; Wallace, R. M.; Kim, M. J.; Mayer, T. S.; Redwing, J. M.; Robinson, J. A. Highly Scalable, Atomically Thin WSe_2 Grown via Metal-Organic Chemical Vapor Deposition. *ACS Nano* **2015**, *9* (2), 2080–2087. https://doi.org/10.1021/nn5073286.

(9) Jmol: an open-source Java viewer for chemical structures in 3D. https://jmol.sourceforge.net/ (accessed 2023-05-15).

(10) Giannozzi, P.; Baroni, S.; Bonini, N.; Calandra, M.; Car, R.; Cavazzoni, C.; Ceresoli, D.; Chiarotti, G. L.; Cococcioni, M.; Dabo, I.; Dal Corso, A.; de Gironcoli, S.; Fabris, S.; Fratesi, G.; Gebauer, R.; Gerstmann, U.; Gougoussis, C.; Kokalj, A.; Lazzeri, M.; Martin-Samos, L.; Marzari, N.; Mauri, F.; Mazzarello, R.; Paolini, S.; Pasquarello, A.; Paulatto, L.; Sbraccia, C.; Scandolo, S.; Sclauzero, G.; Seitsonen, A. P.; Smogunov, A.; Umari, P.; Wentzcovitch, R. M. QUANTUM ESPRESSO: A Modular and Open-Source Software Project for Quantum Simulations of Materials. *J. Phys. Condens. Matter* **2009**, *21* (39), 395502. https://doi.org/10.1088/0953-8984/21/39/395502.

(11) Giannozzi, P.; Andreussi, O.; Brumme, T.; Bunau, O.; Buongiorno Nardelli, M.; Calandra, M.; Car, R.; Cavazzoni, C.; Ceresoli, D.; Cococcioni, M.; Colonna, N.; Carnimeo, I.; Dal Corso, A.; de Gironcoli, S.; Delugas, P.; DiStasio, R. A.; Ferretti, A.; Floris, A.; Fratesi, G.; Fugallo, G.; Gebauer, R.; Gerstmann, U.; Giustino, F.; Gorni, T.; Jia, J.; Kawamura, M.; Ko, H.-Y.; Kokalj, A.; Küçükbenli, E.; Lazzeri, M.; Marsili, M.; Marzari, N.; Mauri, F.; Nguyen, N. L.; Nguyen, H.-V.; Otero-de-la-Roza, A.; Paulatto, L.; Poncé, S.; Rocca, D.; Sabatini, R.; Santra, B.; Schlipf, M.;

Seitsonen, A. P.; Smogunov, A.; Timrov, I.; Thonhauser, T.; Umari, P.; Vast, N.; Wu, X.; Baroni, S. Advanced Capabilities for Materials Modelling with Quantum ESPRESSO. *J. Phys. Condens. Matter* **2017**, *29* (46), 465901. https://doi.org/10.1088/1361-648X/aa8f79.

(12) Giannozzi, P.; Baseggio, O.; Bonfà, P.; Brunato, D.; Car, R.; Carnimeo, I.; Cavazzoni, C.; De Gironcoli, S.; Delugas, P.; Ferrari Ruffino, F.; Ferretti, A.; Marzari, N.; Timrov, I.; Urru, A.; Baroni, S. Quantum ESPRESSO toward the Exascale. *J. Chem. Phys.* **2020**, *152* (15), 154105. https://doi.org/10.1063/5.0005082.

(13) van Setten, M. J.; Giantomassi, M.; Bousquet, E.; Verstraete, M. J.; Hamann, D. R.; Gonze, X.; Rignanese, G.-M. The PseudoDojo: Training and Grading a 85 Element Optimized Norm-Conserving Pseudopotential Table. *Comput. Phys. Commun.* **2018**, *226*, 39–54. https://doi.org/10.1016/j.cpc.2018.01.012.

(14) Hamann, D. R. Optimized Norm-Conserving Vanderbilt Pseudopotentials. *Phys. Rev. B* **2013**, *88* (8), 085117. https://doi.org/10.1103/PhysRevB.88.085117.

(15) Perdew, J. P.; Burke, K.; Ernzerhof, M. Generalized Gradient Approximation Made Simple. *Phys. Rev. Lett.* **1996**, *77* (18), 3865–3868. https://doi.org/10.1103/PhysRevLett.77.3865.

(16) Berland, K.; Cooper, V. R.; Lee, K.; Schröder, E.; Thonhauser, T.; Hyldgaard, P.; Lundqvist, B. I. Van Der Waals Forces in Density Functional Theory: A Review of the vdW-DF Method. *Reports Prog. Phys.* **2015**, *78* (6), 066501. https://doi.org/10.1088/0034-4885/78/6/066501.

(17) Langreth, D. C.; Lundqvist, B. I.; Chakarova-Käck, S. D.; Cooper, V. R.; Dion, M.; Hyldgaard, P.; Kelkkanen, A.; Kleis, J.; Kong, L.; Li, S.; Moses, P. G.; Murray, E.; Puzder, A.; Rydberg, H.; Schröder, E.; Thonhauser, T. A Density Functional for Sparse Matter. *J. Phys. Condens. Matter* **2009**, *21* (8), 084203. https://doi.org/10.1088/0953-8984/21/8/084203.

(18) Lee, K.; Murray, É. D.; Kong, L.; Lundqvist, B. I.; Langreth, D. C. Higher-Accuracy van Der Waals Density Functional. *Phys. Rev. B* **2010**, *82* (8), 081101. https://doi.org/10.1103/PhysRevB.82.081101.

(19) Thonhauser, T.; Zuluaga, S.; Arter, C. A.; Berland, K.; Schröder, E.; Hyldgaard, P. Spin Signature of Nonlocal Correlation Binding in Metal-Organic Frameworks. *Phys. Rev. Lett.* **2015**, *115* (13), 136402. https://doi.org/10.1103/PhysRevLett.115.136402.

(20) Thonhauser, T.; Cooper, V. R.; Li, S.; Puzder, A.; Hyldgaard, P.; Langreth, D. C. Van Der Waals Density Functional: Self-Consistent Potential and the Nature of the van der Waals Bond. *Phys. Rev. B* **2007**, *76* (12), 125112. https://doi.org/10.1103/PhysRevB.76.125112.

(21) Sabatini, R.; Küçükbenli, E.; Kolb, B.; Thonhauser, T.; de Gironcoli, S. Structural Evolution of Amino Acid Crystals under Stress from a Non-Empirical Density Functional. *J. Phys. Condens. Matter* **2012**, *24* (42), 424209. https://doi.org/10.1088/0953-8984/24/42/424209.

(22) Baroni, S.; de Gironcoli, S.; Dal Corso, A.; Giannozzi, P. Phonons and Related Crystal Properties from Density-Functional Perturbation Theory. *Rev. Mod. Phys.* **2001**, *73* (2), 515–562. https://doi.org/10.1103/RevModPhys.73.515.
